# Measuring Patient Value after Total Shoulder Arthroplasty

**DOI:** 10.3390/jcm10235700

**Published:** 2021-12-04

**Authors:** Alexandre Lädermann, Rodolphe Eurin, Axelle Alibert, Mehdi Bensouda, Hugo Bothorel

**Affiliations:** 1 Division of Orthopaedics and Trauma Surgery, La Tour Hospital, 1217 Meyrin, Switzerland; alexandre.laedermann@gmail.com; 2 Faculty of Medicine, University of Geneva, 1211 Geneva, Switzerland; 3 Division of Orthopaedics and Trauma Surgery, Department of Surgery, Geneva University Hospitals, 1205 Geneva, Switzerland; 4 General Management Department, La Tour Hospital, 1217 Meyrin, Switzerland; rodolphe.eurin@latour.ch (R.E.); axelle.alibert@latour.ch (A.A.); mehdi.bensouda@latour.ch (M.B.); 5 Research Department, La Tour Hospital, 1217 Meyrin, Switzerland

**Keywords:** total shoulder arthroplasty, patient reported outcome measures, PROMs, VBHC, value-based health care, patient value, quality, costs

## Abstract

Evaluating the value of health care is of paramount importance to keep improving patients’ quality of life and optimizing associated costs. Our objective was to present a calculation method based on Michael Porter’s formula and standard references to estimate patient value delivered by total shoulder arthroplasty (TSA). We retrospectively reviewed the records of 116 consecutive TSAs performed between June 2015 and June 2019. Patient value was defined as quality of care divided by direct costs of surgery. Quality metrics included intra- and postoperative complications as well as weighted improvements in three different patient-reported outcome measures at a minimum of one-year follow-up. Direct costs of surgery were retrieved from the management accounting analyses. Substantial clinical benefit (SCB) thresholds and the standard reimbursement system were used as references for quality and cost dimensions. A multivariable linear regression was performed to identify factors associated with patient delivered value. Compared to a reference of 1.0, the quality of care delivered to patients was 1.3 ± 0.3 (range, 0.6–2.0) and the associated direct cost was 1.0 ± 0.2 (range, 0.7–1.6). Ninety patients (78%) had a quality of care ≥1.0 and 61 patients (53%) had direct costs related to surgery ≤1.0. The average value delivered to patients was 1.3 ± 0.4 (range, 0.5–2.5) with 91 patients (78%) ≥ 1.0, was higher for non-smokers (beta, 0.12; *p* = 0.044), anatomic TSA (beta, 0.53; *p* < 0.001), increased with higher pre-operative pain (beta, 0.08; *p* < 0.001) and lower pre-operative Constant score (beta, −0.06; *p* = 0.001). Our results revealed that almost 80% of TSAs provided substantial patient value. Patient pre-operative pain/function, tobacco use, and procedure type are important factors associated with delivered patient value.

## 1. Introduction

In the past decades, healthcare challenges have considerably increased due to the global aging of the population and higher treatment costs following advances in medical technologies and medicinal products. In such a context, healthcare actors first focused their interests on reducing costs while giving fewer priorities to patient care quality and efficiency. Therefore, a new disruptive concept emerged to move the current system toward a sustainable and patient-centered model that optimizes both health outcomes and associated costs: the value-based health care (VBHC).

In their work published in 2006, Michael Porter and Elizabeth Teisberg defined value as health outcomes achieved per dollar spent [1]. While this value equation is becoming increasingly prominent, it remains nonetheless difficult to implement in every day clinical practice in absence of a validated method to quantify value and a standard scale for interpretation and benchmarking purposes. In their published article, Reilly et al. [2] proposed an innovative method that allows a surgeon to evaluate the value delivered to his patients after total knee or hip arthroplasties according to the average department results. While we applaud them for this work, the applied methodology relies on the presence of several surgeons for establishing the reference. Moreover, the condition mentioned above can be misleading since it can lead to “false positive” or “false negative” results for a particular surgeon if the entire orthopedic department has low or high outcomes.

With the increasing use of patient-reported outcome measures (PROMs), different thresholds have been published to help understand the amount of PROMs improvement that is clinically relevant to patients [3]. Moreover, the standard direct cost for a specific surgical procedure can be estimated from the national hospital reimbursement system based on diagnosis-related groups (DRG). Therefore, the purpose of the present study was to propose a new calculation method to evaluate the delivered patient value using standard references, thereby shifting the value-based competition from a local orthopedic department to a broader level.

## 2. Materials and Methods

### 2.1. Study Design

The authors retrospectively reviewed the records of consecutive prospectively collected primary TSAs performed at La Tour hospital (Meyrin, Switzerland). The study was conducted according to the Declaration of Helsinki principles, was approved prior to beginning by the Commission cantonale d’éthique de la recherche (CCER) de Genève (Shoulder Outcomes Clinical Study, n° 2014-277), and all patients provided written informed consent for the use of their data for research purposes.

### 2.2. Patient Selection, Demographic and Operative Data

**Figure 1:**
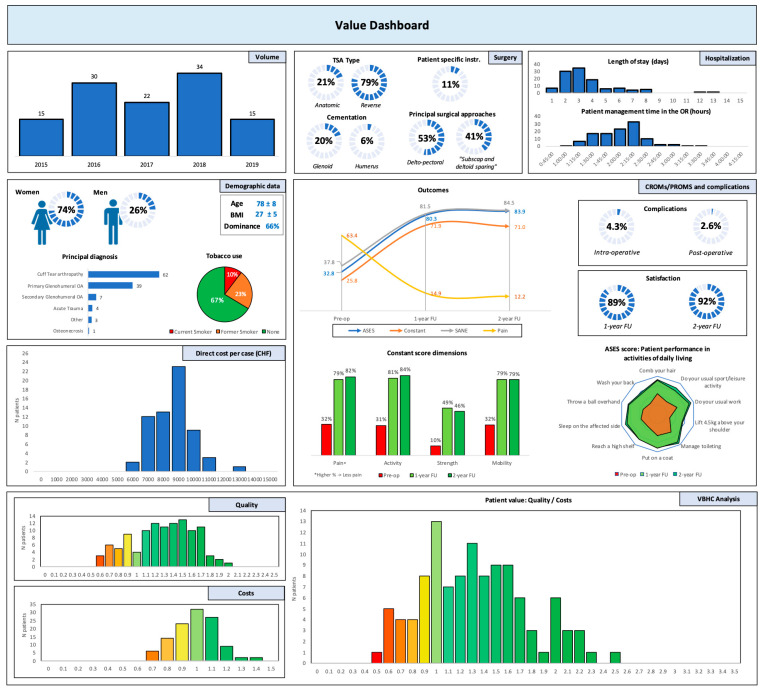
Value dashboard.

Between June 2015 and June 2019, 284 patients had a primary shoulder arthroplasty performed by the senior author (A.L.). Patients were included in the study if they were operated on at La Tour hospital and underwent a TSA (*n* = 139). Patients were excluded if they did not have a complete pre-operative evaluation due to the need for emergency care (*n* = 13), if they deceased due to other reasons than surgery before the 1-year follow-up visit (*n* = 5), and if they were lost to follow-up (LTFU, *n* = 6). This left a study cohort of 116 patients aged 77.8 ± 7.6 years (median, 78.0; range, 57–94) at index surgery, comprising 86 women (74%) and 30 men (26%), available for analyses (Table 1, Figure 1). The principal diagnoses were rotator cuff tear arthropathy (*n* = 62, 53%), primary glenohumeral osteoarthrosis (*n* = 39, 34%), secondary glenohumeral osteoarthrosis (*n* = 7, 6%), acute trauma (*n* = 4, 3%), osteonecrosis (*n* = 1, 1%), and others in 3 cases (3%). The type of procedure was anatomic TSA for 24 patients (aTSA, 21%) and reverse TSA for the other 92 patients (rTSA, 79%). The operations were performed by a single senior surgeon (A.L.). A majority of the patients were operated on the dominant arm (*n* = 77, 66%) through a deltopectoral approach (*n* = 62, 53%) or subscapularis and deltoid sparing approach (*n* = 47, 41%) [4,5]. A patient specific guide was used in 13 cases (11%) to help the prothesis implantation and cementation was required in 23 cases on the glenoid side (20%, for aTSA only) and in 7 cases on the humeral side (6%). The patient management time in the operating room (OR) was 121 ± 26 min (median, 125; range, 60–210 min) and patient length of stay was 3.6 ± 2.0 days (median, 3.0; range, 1.2–12.9 days).

### 2.3. Study Variables

The outcome of interest was the delivered patient value. The data analyzed in this study comprised the characteristics of the patient (age, gender, BMI, arm dominance, principal diagnosis), surgery (anatomic/reverse TSA, approach, cementation, patient management time in the operating room, intraoperative complications), hospitalization (length of stay, direct cost), complications, patient satisfaction, and PROMs.

### 2.4. Clinical Evaluation

Patients were evaluated at a minimum follow-up of one year (59 at 1 year and 57 at 2 years). The clinical outcomes included periprosthetic joint infection (PJI), implant revision, and other intra- or postoperative complications. The PROMs included the American Shoulder and Elbow Surgeons (ASES) score [6], the Constant score [7], the Single Assessment Numeric Evaluation (SANE) score [8,9], and the pain on a visual analogue scale (VAS). The ASES score (from 0 worst to 100 best) comprises one pain item and 10 questions relative to patient function/disability. The Constant score (from 0 worst to 100 best) has four different dimensions, including pain, activity, strength, and mobility. The SANE score (from 0 worst to 100 best) was assessed with a single question: “How would you rate your affected shoulder today as a percentage of normal (0% to 100% scale with 100% being normal)” and the pain on VAS (from 0 best to 10 worst) was rescaled to a range of 0–100 points. The reference used for each PROM improvement was the substantial clinical benefit calculated by Simovitch et al. in a combined cohort of 1856 reverse and anatomic TSA (31.5 points for the ASES score, 19.1 points for the Constant score, 32 points for pain on VAS) [3]. Although being a useful metric, we did not include the SANE score improvement in the quality evaluation since its SCB has not been robustly validated in the scientific literature yet [10]. Furthermore, the SANE score has been reported to be moderately/strongly correlated with the ASES score [11]. The authors also used the minimal clinically important differences (MCID) for the aforementioned scores for descriptive analyses (13.6 points for the ASES score, 5.7 points for the Constant score, 16 points for pain on VAS) [3].

### 2.5. Equation for Patient Value

The equation used for the calculation of patient value was based on the one previously published by Reilly et al. [2]:

$$\mathrm{Patient}\;\mathrm{value}=\frac{\mathrm{Quality}}{\mathrm{Cost}}=\frac{\left(\mathrm{Weighted}\;\mathrm{clinical}\;\mathrm{outcomes}+\mathrm{Weighted}\;\mathrm{PROMs}\;\mathrm{improvement}\right)}{\begin{array}{c} \mathrm{Direct}\;\mathrm{cost} \end{array}}$$

The value equation can therefore be written as follows:

$$Value=\frac{W_{1}\left(\frac{\Delta P_{ASES}}{SCB_{ASES}}\right)+W_{2}\left(\frac{\Delta P_{Constant}}{SCB_{Constant}}\right)+W_{3}\left(\frac{\Delta P_{VAS\;Pain}}{SCB_{VAS\;Pain}}\right)+W_{4}\left(P_{Intra-op\;Comp}\right)+W_{5}\left(P_{Post-op\;Comp}\right)+W_{6}\left(P_{P\;JI}\right)+W_{7}\left(P_{revision}\right)}{\left(\frac{P_{direct\;\mathrm{cost}}}{DRG_{direct\;\mathrm{cost}}}\right)}$$

As detailed in the article of Reilly et al. [2], all negative pre- to postoperative improvements were forced to 0, and clinical outcomes were coded as binary depending on the event occurrence. The absence of event resulted in a patient score equaling 0 for that outcome, while the presence of it resulted in a patient score equaling the total weight. The weighting for the clinical outcomes and PROMs was performed by the senior surgeon (AL) according to his strong clinical experience and scientific knowledge. For clarity, a quality of 1.0 indicated an improvement in PROMs which was equal to the defined SCBs and an absence of any complication, PJI or revision. A cost of 1.0 indicated a TSA that cost the exact direct cost reference (see below). The result of the equation (quality/cost) was rounded at the first decimal place and indicated a substantial delivered value if ≥ 1.0 or an unsubstantial delivered value if < 1.0.

### 2.6. Costs

The cost was defined as the direct cost related to the surgical procedure (material and medicine costs only). This data was exported from the management accounting REKOLE^®^ analyses that are performed annually at our institution. The standard reimbursement for a TSA was calculated by multiplying the hospital base rate (9550 CHF) by the DRG standard cost-weight, which varied between 1.929 and 2.096 from 2015 to 2019. The standard TSA reimbursement for a patient with basic insurance only was therefore 19,081 CHF in 2015, 20,017 CHF in 2016, 18,651 in 2017, 18,422 CHF in 2018, and 18,479 CHF in 2019. In our consecutive series of patients with basic insurance only (*n* = 47, 38%), the direct cost per case represented 44% of the total cost (44% ± 7%; median, 44%; range, 32–60%). Thus, we considered that 44% of the standard TSA reimbursement should be used as the direct cost reference, which gives: 8396 CHF in 2015, 8807 CHF in 2016, 8206 in 2017, 8106 CHF in 2018, and 8131 CHF in 2019.

### 2.7. Statistical Analyses

For baseline characteristics, variables were reported as mean ± standard deviation or proportions. Shapiro–Wilk tests were used to assess the normality of distributions. Differences between preoperative and postoperative continuous values were evaluated using either the paired Student’s t-test (if Gaussian distribution) or the Wilcoxon signed-rank test (if non-Gaussian distribution). The correlation between the quality and cost was analyzed using the Pearson’s coefficient. A multivariable linear regression model was performed to identify which pre-operative factors (Constant and ASES scores, VAS pain, primary diagnosis), patient characteristics (age, gender, BMI, arm dominance, and tobacco use), and intra-operative factors (patient management time in the operating room, surgical procedure, surgical approach, cementation and use of patient specific instrumentation) were independently associated with patient delivered value.

The variables included in the multivariable regression model were identified using the backward selection method with a threshold of significance set at a *p* value < 0.05 (pre-operative Constant score, pre-operative VAS pain, tobacco use, and surgical procedure). Statistical analyses were performed using R version 3.6.2 (R Foundation for Statistical Computing, Vienna, Austria), and *p*-values < 0.05 were considered statistically significant.

## 3. Results

### 3.1. Clinical Outcomes

The weighting for the clinical outcomes were as follows: 0.1 for the ASES and Constant scores, 0.2 for the VAS pain, 0.1 for an intra- or postoperative complication, and 0.2 for a PJI or an implant revision.

From the final cohort of 116 patients, 5 (4.3%) had an intra-operative complication and 17 (15%) experienced a postoperative complication (Table 2). Three patients (3.4%) underwent an implant revision within the 2 postoperative years due to a component dislocation (revised at 2 months), an implant dissociation (revised at 2 months), and a humeral implant loosening (*n* = 1 revised at 6 months). It is worth noting that none of our patients had a PJI, and that patient satisfaction was 89% at 1-year follow-up and 92% at 2-year follow-up.

At their last follow-up (1.5 ± 0.5 years), our patients significantly improved their VAS pain (49 ± 29 points) as well as SANE (45.1 ± 25.6 points), Constant (45.2 ± 20.2 points), and ASES scores (48.2 ± 23.8) (Table 3). The MCID threshold for the Constant score, ASES score, and VAS pain was achieved by 97%, 89%, and 83% of the patients, while the SCB threshold for similar scores was, respectively, reached by 88%, 77%, and 68% of the cases.

### 3.2. Costs

The total cost per case was 17,954 ± 3383 CHF (median, 17,433; range, 12,757–27,517 CHF) with an average direct cost of 8311 ± 1243 CHF (median, 8442; range, 5347–12,849 CHF).

### 3.3. Patient Value

**Figure 2:**
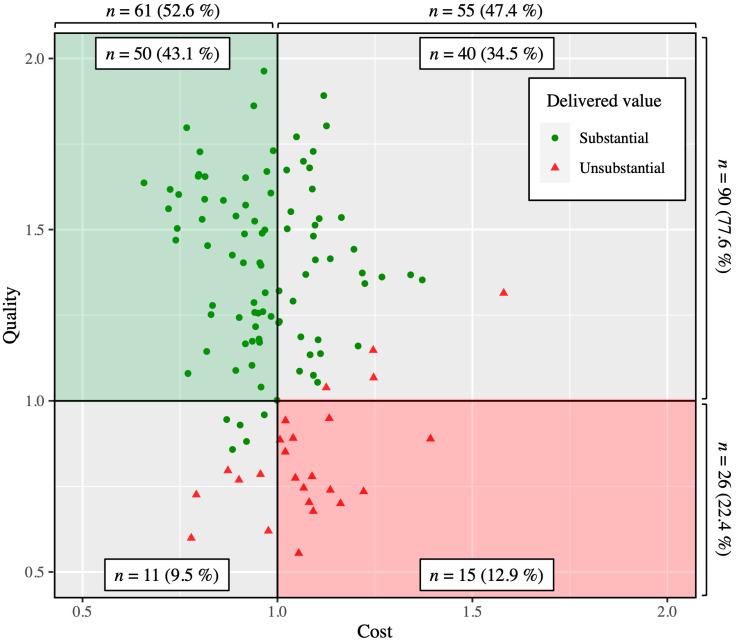
Scatter plot illustrating cost versus quality measures with patient delivered value.

According to the patient value equation presented in the methods section, the quality of care delivered to patients was 1.3 ± 0.3 (median, 1.3; range, 0.6–2.0), and the associated cost was 1.0 ± 0.2 (median, 1.0; range, 0.7–1.6). Ninety patients (78%) had a quality of care ≥1.0 and 61 patients (53%) had a direct cost related to surgery ≤1.0 (Figure 2). No significant correlation was found between cost and quality (*r* = −0.17, CI = −0.34–0.02; *p* = 0.076). Considering these two dimensions, the average value delivered to patients was 1.3 ± 0.4 (median, 1.3; range, 0.5–2.5), with 91 patients (78%) equaling or exceeding 1.0 (Figure 1). Among the 55 patients with a cost >1.0, 36 (65%) had still a substantial delivered value owing to a high quality of care. Likewise, among the 26 patients with a quality of care <1.0, 5 (19%) had a substantial delivered value thanks to a lower cost than expected. The multivariable linear regression revealed that patient delivered value was significantly higher for non-smokers (beta, 0.12; 95%CI, 0.00–0.23; *p* = 0.044), patients operated with anatomic TSA (beta, 0.53; 95%CI, 0.39–0.66; *p* < 0.001), increased with higher (worse) pre-operative VAS pain (beta for 10 points of VAS pain, 0.08; 95%CI, 0.06–0.11; *p* < 0.001) but reduced with higher pre-operative Constant score (beta for 10 points of Constant score, −0.06; 95%CI, −0.03–−0.10; *p* = 0.001).

## 4. Discussion

Evaluating the value of health care is of paramount importance to keep improving patients’ quality of life and optimizing associated costs. Hospitals’ digitalization is still ongoing and offers a great potential for patients’ evaluation along their entire care path. Beyond this, the real challenge that often arises in VBHC discussions is the absence of external benchmarks which compels us to compare our results within our institution or at different time intervals. The authors of the present study therefore created a new value-based dashboard for TSA, which allows an objective comparison with standard references.

According to our results, 78% of the TSAs performed at our institution offered a substantial value to patients. It is worth noting that 41 patients (35%) had a substantial delivered value although they had either a quality of care below the expectations or an excessive direct cost (Figure 2). This emphasizes the importance of evaluating both indicators together rather than interpreting them independently from each other.

Different authors recently evaluated the value delivered by TSA at short term using different methods [12,13,14]. Menendez et al. [14] defined the delivered value as the postoperative ASES score divided by the hospitalization time-driven activity-based costs. More comparable to our value calculation method, Berglund et al. [13] divided the ratio of PROM improvement (in units of MCID) by the total hospitalization cost. Both aforementioned studies found that reverse TSA was associated with a decreased delivered value compared to anatomic TSA, which corroborates our findings. Although it was expected given that reverse TSA has a higher cost associated with the management of rotator cuff deficiencies, it is important to note that such an association can be reversed at some point since different studies already revealed mid- or long-term concerns on anatomic TSA (glenoid loosening, difficult revision procedures, and disappointing outcomes) [15,16,17]. Furthermore, the indications for these two procedures can be different and further analyses with matched cohorts are needed [18].

In our study, the delivered value was higher for shoulders with a lower preoperative function or higher pre-operative pain since greatest clinical improvements are usually observed for patients with worse preoperative health [19]. Our analyses also revealed that current or former smokers had a lower delivered value compared to non-smokers. The negative impact of tobacco use on outcomes after TSA is well reported [20,21,22,23] and emphasizes smoking cessation programs [24]. In the next decades, machine learning algorithms might be able to accurately predict postoperative patient outcomes based on their preoperative characteristics [25]. Such prognostic tools would help manage patient expectations [26] and avoid surgery for patients who would not benefit from it, thereby reducing associated risks for the patients while lowering costs for the health care system.

Different authors already worked on the creation of VBHC dashboards/scorecards [2,27]. Riley et al. published an innovative method to illustrate patient value following total hip and knee arthroplasties [2]. This method consisted of comparing the results of different surgeons within the same institution, which motivates them to outperform for the sake of their patients. However, the use of internal references such as orthopedic department averages for direct costs or PROMs can be misleading. For instance, implementing this method in small institutions where only one surgeon works in a specific medical field would be unwarranted. Furthermore, this method could reveal outstanding results for a surgeon even though the entire department has bad outcomes. In our study, we proposed to use SCB thresholds for the interpretation of PROMs improvements and to estimate the direct cost reference by using the DRG-based standard reimbursement system. The proposed dashboard can guide toward patient value improvement before a new methodology and strong external benchmarks using data from several hospitals are created.

Continuous improvements based on measuring the own performance in order to provide the best possible value to customers has been a key success factor for successful companies across all industries. VBHC is bringing this principle into health care, to the great benefit of patients and the system. The mentality of the different health care players is changing, and the competition slowly shifts from micro-costing only to patient outcome and cost optimization. It is setting the stage for a new way of thinking, collaborating, and competing, thereby opening new opportunities to reinforce excellence in care. The combination of medical expertise with an open mindset for change and self-evaluation is essential. In this sense, VBHC is redefining the basis of what leadership is for healthcare professionals. An essential development will be the emergence of new reimbursement models rewarding better outcomes. This will again require a fundamental change in people’s mindset, while providing a great opportunity for early adopters to accelerate change.

Our study has several limitations. First, our analyses only illustrate the delivered patient value at short follow-up. Furthermore, patients for whom complications were noted might have been double-penalized since such clinical outcomes might also affect PROMs. To reduce the aforementioned bias, an artificial floor was used for patients who had a negative change in PROMs. Second, the weighting of clinical outcomes was solely based on the senior surgeon’s experience. The logic was to attribute an equal weight (0.2) to the five principal outcomes that are crucial for shoulder arthroplasty success: global function (ASES and Constant scores), pain, complication (intra- or post-operatively), PJI, and revision. If a principal outcome comprised different sub-outcomes (e.g., Global function), the weight was then split proportionally to have a similar weighting between sub-outcomes (e.g., 0.1 for the ASES score and 0.1 for the Constant score). A Delphi method engaging the patients, insurance providers, and other key important players would have been more appropriate. Third, our outcome and cost indicators were not risk-adjusted, which can represent a bias if comparisons are made between two surgeons with differences in case mix and patient populations. Fourth, the direct cost reference was estimated to be 44% of the standard TSA reimbursement based on our patients with basic insurance only. A thorough analysis of the DRG-based standard reimbursement system should be performed and published so that each institution knows the theoretical amount supposed to cover direct costs. Fifth, the MCID and SCB values might change across different patient populations. Lastly, a broader analysis focusing on a specific pathology (e.g., glenohumeral arthritis) rather than on a particular treatment (e.g., TSA) would be more in line the VBHC concept.

## 5. Conclusions

The proposed calculation method provides an estimation of delivered patient value using standard references. Such a dashboard could be used to implement VBHC in everyday clinical practice. Our results revealed that TSAs performed at our institution provided substantial patient value in almost 80% of our cases. Patient pre-operative pain/function, tobacco use, and type of procedure (anatomic or reverse) are important factors associated with patient value after TSA. A VBHC community gathering all the different key players is definitely needed to establish solid guidelines and improve our practice according to experiences of each.

## Figures and Tables

**Table 1:** Pre- and intra-operative data.

	Final Cohort (*n* = 116 Patients)			
	*n*	(%)		
	Mean	±SD	Median	(Range)
Preoperative data				
Age	77.8	±7.6	78.0	(57.0–94.0)
Body mass index	27.4	±4.8	26.7	(17.6–42.8)
Male gender	30	(25.9%)		
Principal diagnosis				
Rotator cuff tear arthropathy	62	(53.4%)		
Primary glenohumeral osteoarthrosis	39	(33.6%)		
Secondary glenohumeral osteoarthrosis	7	(6.0%)		
Acute trauma	4	(3.4%)		
Osteonecrosis	1	(0.9%)		
Others	3	(2.6%)		
Dominant arm affected	77	(66.4%)		
Intraoperative data				
Surgical procedure				
Anatomic Total Shoulder Arthroplasty (aTSA)	24	(20.7%)		
Reverse Total Shoulder Arthroplasty (rTSA)	92	(79.3%)		
Surgical approach				
Deltopectoral	62	(53.4%)		
Subscapularis and deltoid sparing	47	(40.5%)		
Anterior Deltoid Detachment with Lateral Split	3	(2.6%)		
Subscapularis sparing	3	(2.6%)		
Transdeltoid	1	(0.9%)		
Use of patient specific instrumentation				
Software (planification)	116	(100.0%)		
Hardware (guide)	13	(11.2%)		
Cementation				
Humeral side	7	(6.0%)		
Glenoid side	23	(19.8%)		

**Table 2:** Intra and post-operative complications.

	Final Cohort (*n* = 116 Patients)	
	*n*	(%)
Intraoperative complications	5	(4.3%)
Unplanned humeral fractures	5	(4.3%)
Postoperative complications	17	(14.7%)
Acromial fracture	7	(6.0%)
Component loosening	2	(1.7%)
Deltoid Muscle Dysfunction	2	(1.7%)
Instability-Dislocation	2	(1.7%)
Component dissociation	1	(0.9%)
Instability-Subluxation	1	(0.9%)
Rotator Cuff Tear	1	(0.9%)
Nerve Palsy (other than axillary)	1	(0.9%)
Implant revisions	3	(2.6%)

**Table 3:** Pre- and post-operative outcomes.

	Preoperative Status				Postoperative Status (Last Follow-Up)				Absolute Improvement			
	Mean	±SD	Median	(Range)	Mean	±SD	Median	(Range)	Mean	±SD	Median	(Range)
SANE score	37.6	±22.2	30.0	(0.0–90.0)	82.4	±16.9	90.0	(20.0–100.0)	45.1	±25.6	45.0	(0.0–100.0)
Constant score	25.7	±15.0	24.0	(0.0–62.4)	70.8	±16.4	74.2	(24.0–99.2)	45.2	±20.2	47.1	(0.0–83.0)
Strength	2.4	±4.3	0.0	(0.0–17.6)	11.5	±6.2	11.0	(0.0–25.0)	9.7	±6.4	9.9	(0.0–25.0)
Mobility	12.8	±10.7	10.0	(0.0–40.0)	31.5	±7.3	32.0	(8.0–40.0)	18.6	±11.6	19.5	(0.0–40.0)
Pain	4.8	±3.2	4.0	(0.0–15.0)	12.0	±3.8	14.0	(0.0–15.0)	7.3	±4.3	7.0	(0.0–15.0)
Activity	6.2	±3.5	6.0	(0.0–15.0)	16.1	±4.3	18.0	(0.0–20.0)	10.0	±5.3	10.0	(0.0–20.0)
ASES score	32.6	±16.2	32.5	(0.0–82.0)	81.1	±19.8	87.0	(13.0–100.0)	48.2	±23.8	50.0	(0.0–100.0)
Pain	18.5	±11.4	15.0	(0.0–50.0)	42.6	±10.1	45.0	(10.0–50.0)	24.5	±14.4	25.0	(0.0–50.0)
Activity	14.1	±8.6	13.0	(0.0–42.0)	38.4	±11.9	43.0	(3.0–50.0)	24.4	±12.8	26.5	(0.0–50.0)
VAS Pain *	64	±22	70	(0–100)	15	±20	10	(0–80)	49	±29	50	(0–100)

SANE, Single Assessment Numeric Evaluation; ASES, American Shoulder and Elbow Surgeons; VAS, Visual Analogue Scale; * A decrease in VAS Pain indicates a good result. A positive improvement is noted if the VAS Pain decreases. All pre- versus post-operative scores were statistically significant (*p* < 0.001).

## Data Availability

Details regarding where data supporting reported results can be requested at the following e-mail address: hugo.bothorel@latour.ch.
